# Acquisition and execution of motor sequences by a computational model of the cerebellum

**DOI:** 10.1186/1471-2202-14-S1-P409

**Published:** 2013-07-08

**Authors:** Ivan Herreros, Santiago Brandi, Paul FMJ Verschure

**Affiliations:** 1 Specs, Unversitat Pompeu Fabra, Barcelona, Spain; 2 Institució Catalana de Recerca i Estudis Avaçats (ICREA), Barcelona, Spain

## 

Although there is evidence that the cerebellum is sufficient for the execution of certain action sequences [1], its role in motor sequence acquisition is still unclear. Motor sequence acquisition is studied with complex tasks (e.g., serial reaction-time) that involve several brain areas, such as the cortex and the striatum, in addition to the cerebellum. Due to these interactions it is hard to disentangle the specific function performed by the cerebellum alone. With a computational study, here we address whether the cerebellum alone is sufficient for the learning of action sequences in a simple motor acquisition task.

To achieve this we extend an existing cerebellar-based control architecture [2] by adding the Nucleo-Pontine Projections (NPPs). Such NPPs establish the so-called cerebello-pontine loops, such that cerebellar output at a given time can be fed back to the cerebellar input layer. Hypothetically, given the recurrence established by the NPPs, the cerebellum could control action sequences, chaining the next action execution to the current one.

We test this prediction with a variation of the classical conditioning paradigm, where a Conditioning Stimulus (CS) is followed not just by a single Uncoditioned Stimulus (US), but by multiple USs. Provided that the inter-stimulus intervals are sufficiently long, only the first Conditioned Response (CR) should be triggered by the CS and all the following CRs should be recurrently triggered.

To confirm that learning in the cerebellum is sufficient to acquire action sequences within the above-mentioned paradigm, we apply our model to two different scenarios: in the first, the model acquires a series of CRs in the classical conditioning of the eye-blink, a paradigm commonly used to study learning in the cerebellum; whereas in the second, the model controls a real robot that traverses a track with multiple turns. In the latter scenario, collision with walls acts as the US and the visual stimulus is the CS. After learning, the CS alone triggers a sequence of well-timed turns.

This work leads to two applications. On one hand, we demonstrate a control system that can autonomously acquire motor responses encompassing multiple linked actions. This system is strongly based in the known cerebellar circuitry and can be embedded in robotic platforms. On the other hand, these results provide a set of experimental predictions that can guide the study of cerebellar function in the acquisition of action sequences.
